# Reconditioning the Neurogenic Niche of Adult Non-human Primates by Antisense Oligonucleotide-Mediated Attenuation of TGFβ Signaling

**DOI:** 10.1007/s13311-021-01045-2

**Published:** 2021-04-15

**Authors:** Sebastian Peters, Sabrina Kuespert, Eva Wirkert, Rosmarie Heydn, Benjamin Jurek, Siw Johannesen, Ohnmar Hsam, Sven Korte, Florian Timo Ludwig, Lars Mecklenburg, Heike Mrowetz, Barbara Altendorfer, Rodolphe Poupardin, Susanne Petri, Dietmar R. Thal, Andreas Hermann, Jochen H. Weishaupt, Joachim Weis, Inci Sevval Aksoylu, Sebastian A. Lewandowski, Ludwig Aigner, Tim-Henrik Bruun, Ulrich Bogdahn

**Affiliations:** 1 Department of Neurology, University Hospital Regensburg, Regensburg, Germany; 2 Velvio GmbH, Am Biopark 11, Regensburg, Germany; 3 Institute for Molecular and Cellular Anatomy, University of Regensburg, Regensburg, Germany; 4 Covance Preclinical Services GmbH, Muenster, Germany; 5 Institute of Molecular Regenerative Medicine, Spinal Cord Injury and Tissue Regeneration Center Salzburg (SCI-TReCS), Paracelsus Medical University Salzburg, Salzburg, Austria; 6 Institute of Experimental and Clinical Cell Therapy, Spinal Cord Injury and Tissue Regeneration Center (SCI-TReCS), Paracelsus Medical University Salzburg, Salzburg, Austria; 7 Department of Neurology, University Hospital MHH, Hannover, Germany; 8 Department for Imaging and Pathology, Laboratory for Neuropathology, University of Leuven, Leuven, Belgium; 9 Laboratory of Neuropathology, Institute of Pathology, Ulm University, Ulm, Germany; 10 Translational Neurodegeneration Section „Albrecht-Kossel”, Department of Neurology, University Medical Center Rostock, University of Rostock, and German Center for Neurodegenerative Diseases (DZNE) Rostock, Rostock, Germany; 11 Department of Neurology, University Hospital Mannheim, Mannheim, Germany; 12 Institute of Neuropathology, RWTH Aachen University Medical School, Aachen, Germany; 13 Department of Clinical Neuroscience, Center for Molecular Medicine, Karolinska Institute, Stockholm, Sweden; 14 SciLifeLab, School of Biotechnology, Royal Institute of Technology, Stockholm, Sweden

## Abstract

Adult neurogenesis is a target for brain rejuvenation as well as regeneration in aging and disease. Numerous approaches showed efficacy to elevate neurogenesis in rodents, yet translation into therapies has not been achieved. Here, we introduce a novel human TGFβ-RII (Transforming Growth Factor—Receptor Type II) specific LNA-antisense oligonucleotide (“locked nucleotide acid”—“NVP-13”), which reduces TGFβ-RII expression and downstream receptor signaling in human neuronal precursor cells (ReNcell CX® cells) *in vitro*. After we injected cynomolgus non-human primates repeatedly i.th. with NVP-13 in a preclinical regulatory 13-week GLP-toxicity program, we could specifically downregulate TGFβ-RII mRNA and protein *in vivo*. Subsequently, we observed a dose-dependent upregulation of the neurogenic niche activity within the hippocampus and subventricular zone: human neural progenitor cells showed significantly (up to threefold over control) enhanced differentiation and cell numbers. NVP-13 treatment modulated canonical and non-canonical TGFβ pathways, such as MAPK and PI3K, as well as key transcription factors and epigenetic factors involved in stem cell maintenance, such as MEF2A and pFoxO3. The latter are also dysregulated in clinical neurodegeneration, such as amyotrophic lateral sclerosis. Here, we provide for the first time *in vitro* and *in vivo* evidence for a novel translatable approach to treat neurodegenerative disorders by modulating neurogenesis.

**Supplementary Information:**

The online version contains supplementary material available at 10.1007/s13311-021-01045-2.

## Introduction

Intact neurogenesis is key to constant brain and spinal cord (SC) rejuvenation, with newborn neurons integrating into individual neural networks throughout lifetime, thus reacting to challenges with structural and functional adaptations. Showing that neurogenesis is essential for this process was first reported by Altman et al. [1], in lesioned rat brains, and by Erikson et al. who first described human neurogenesis within hippocampi of post-mortem adult cancer patients [2]. Regions of mammalian neurogenesis comprise the hippocampal subgranular zone of the dentate gyrus (SGZ) and the subventricular zone (SVZ) of the lateral ventricle [3, 4]. In addition to other regions of the central nervous system (CNS [5–10]), the SC of rodents [11, 12] and primates [13] has also been described as critical regions for neurogenesis. Neurogenic niche activity is compromised in chronic neurodegenerative disorders such as amyotrophic lateral sclerosis (ALS) [14], and even though clinical relevance of adult human neurogenesis to date is controversial [15-17], its stimulation for cell renewal reactivation may prompt new treatment horizons.

The transforming growth factor β (TGFβ) system is a powerful regulator of neurogenic niche activity controlling proliferation, cell differentiation, and stem cell activity. Upregulated TGFβ signaling drives fibrosis of the EM (extracellular matrix), induces dysfunctional autophagy in neurons, and induces a broad spectrum of systemic and local immune dysfunctions [18–22]. TGFβ activates SMAD (also known as mothers against DPP homolog 1)-dependent (canonical) and SMAD-independent (non-canonical) signaling cascades through receptor subtypes I and II [23, 24]. Depending on local context, dose, and exposure times [25–27], effects of TGFβ signaling may be either beneficial (neuroprotective, activating stem cells, anti-inflammatory, inducing autophagy) or detrimental (neurodestructive, arresting stem cell renewal, pro-inflammatory, pro-fibrotic, inhibitory for autophagy) [19, 28, 29]. Due to the chronic systemic and local neuroinflammatory milieu, neurodegenerative disorders exhibit an upregulated TGFβ-system. We could recently show that compared to healthy controls, TGFβ and its receptor TGFβ-RII are upregulated in post mortem CNS tissue of ALS patients [14] and that stem cell niche activity is subsequently decreased in the brain and SC. A very recent review highlighted the TGFβ-system to be altered and crucially involved in the disease course in ALS, with dysfunctional signaling in early stages and a persistent over-activation at the clinical stage of disease [30].

To follow up the hypothesis of a dysregulated TGFβ-system underlying the detrimental disease course of neurodegenerative disorders like ALS, we developed a novel antisense oligonucleotide (NVP-13) in a locked nucleic acid (LNA)-gapmer design, targeting TGFβ-RII mRNA. With NVP-13 treatment, reducing the ligand binding TGF-RII protein, we aimed to reverse various pathological hallmarks of neurodegeneration, including reduced neurogenesis, in a highly efficient and safe manner [31] ready for clinical translation. The goals of the current study were (i) to determine the efficacy of NVP-13 on TGFβ mRNA and protein downregulation *in vivo*, (ii) to further characterize changes in downstream phosphorylation of intracellular signaling cascades, (iii) to investigate the regulation of neurogenic niche marker expression both *in vitro* (human neural precursor cells) and *in vivo* (male and female cynomolgus monkeys), and (iv) to evaluate potential clinical relevance of NVP-13 treatment.

## Results

### NVP-13 Significantly Downregulates TGFβ-RII mRNA and Protein Expression *In Vitro*

**Fig. 1:**
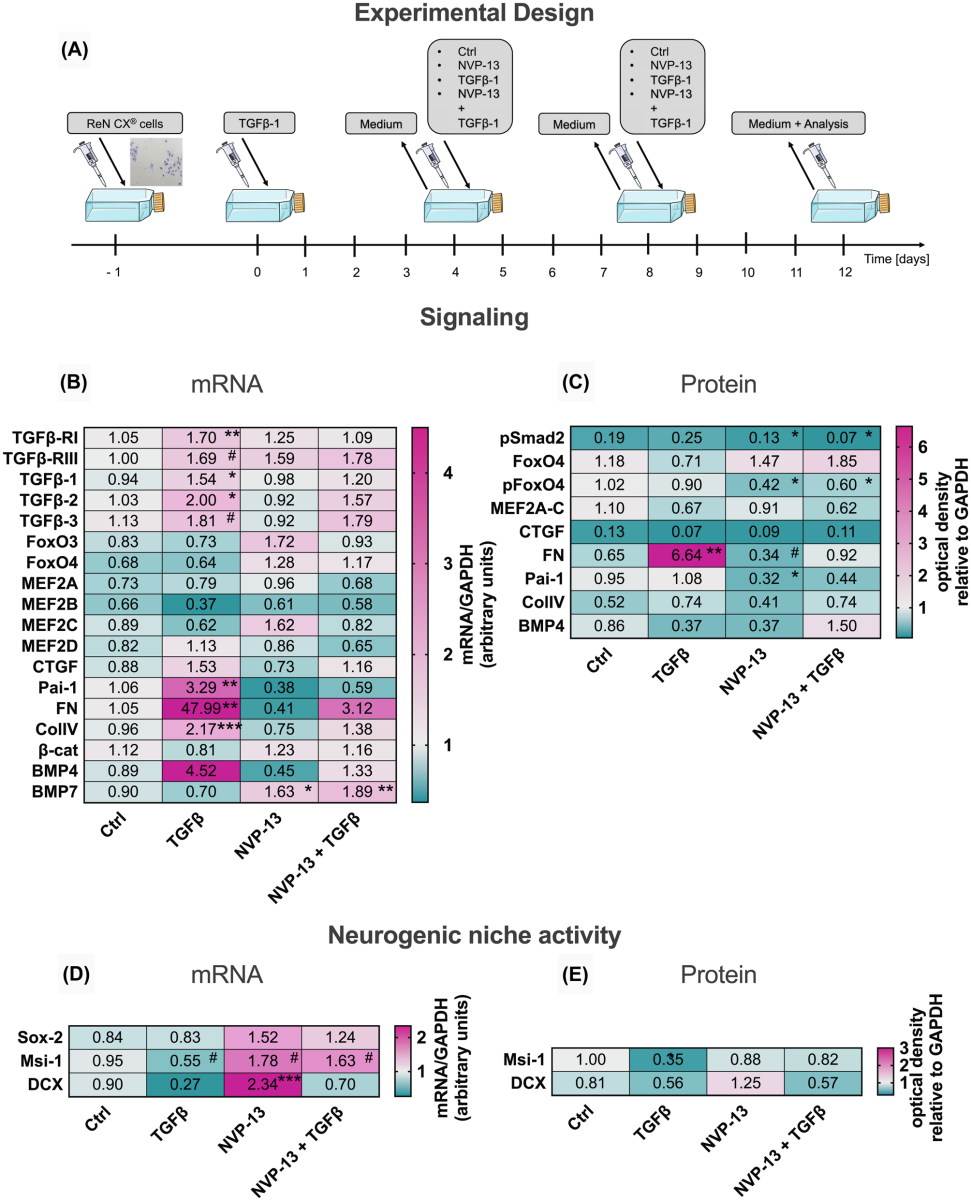
*In vitro* effects of NVP-13 on target regulation, intracellular pathways, and neurogenic niche markers. Human neural progenitor cells (ReNcell CX® cells) were cultured according to manufacturer’s recommendations. Cells were pretreated with TGFβ1 (50 ng) to simulated diseased conditions. Following 4 days of pretreatment, TGFβ1-containing medium was removed, and cells were cultured in fresh medium containing either PBS (control, *n* = 7/8), NVP-13 (10 µM, *n* = 6/8), TGFβ1 (50 ng/ml, *n* = 7/8), or a combination of NVP-13 (10 µM) and TGFβ1 (50 ng/ml, *n* = 5/8). This procedure was repeated on experimental day 8. On experimental day 12, the medium was removed, and the cells were harvested for mRNA, protein, and immunofluorescence analysis **(A)**. TGFβ1 treatment resulted in an activated system indicated by enhanced mRNA expression levels of receptor subtype I, ligand 1, 2, Pai-1, Fibronectin, Collagen IV, and a tendency towards enhanced mRNA expression levels of receptor subtype III and ligand 3. mRNA expression levels of BMP7 were reduced for both NVP-13 groups and unaltered for TGFβ1-treated cells compared to controls **(B)**. NVP-13 resulted in reduced phosphorylation of TGFβ signaling molecules Smad2 and FoxO4 (NVP-13 alone and cotreatment with TGFβ1). Further, under uncombined conditions, NVP-13 significantly reduced the protein expression of Pai-1 **(C)**. mRNA expression levels of Msi-1 were opposed with a tendency towards enhanced levels for both NVP-13 groups and a tendency towards decreased expression for the TGFβ1-treated cells compared to controls **(D)**. As expected, TGFβ1 incubation significantly reduced Msi-1 protein expression, which was ameliorated by NVP-13 **(E)**. All parameters were tested for Gaussian distribution using the Shapiro–Wilk normality test (samples sizes too small for D´Augostino-Pearson omnibus normality test). Afterwards, all parameters were analyzed using a one-way ANOVA followed by Tukey’s post hoc test or a Kruskal-Wallis test followed by Dunn’s post hoc test, depending on Gaussian distribution. Data are presented as median with min to max (box plots) and median (heat maps). Significance was taken at *p* ≤ 0.05 and a trend was noted at *p* ≤ 0.1. #*p* < 0.1 vs 0.9% NaCl (Trend); *p < 0.05; **p < 0.01; ***p < 0.001; ****p < 0.0001 vs 0.9% NaCl (significant)

To evaluate *in vitro* efficacy, human neural precursor cells (ReNcell CX® cells) were pre-treated with TGFβ1 (50 ng/ml, to simulate the neuroinflammatory process) for 4 days. Then, cells were either treated with phosphate-buffered saline (PBS; as control), NVP-13 (10 µM), TGFβ1 (50 ng ligand concentration simulating an unchanged concentration in the pathology *in vivo*), or NVP-13 + TGFβ1 (10 µM + 50 ng) (Fig. 1A). Data have been published recently in detail [31] showing that NVP-13 significantly downregulates TGFβ-RII mRNA (qPCR) and protein (WB) expression *in vitro*, which was confirmed by immunocytochemistry under physiological and also disease-simulating conditions. To mimic the neuroinflammatory process characterized by increased TGFβ-system activity, TGFβ co-incubation was always investigated in parallel. Next, we analyzed TGFβ signaling components that mediate typical downstream effects, including neurogenesis and extracellular matrix formation.

### Targeting TGFβ-RII mRNA with NVP-13 Modulates Intracellular Fibrosis and Stem Cell Niche Markers Towards a More Stem Cell Favoring Milieu

Treatment of human neural precursor cells with TGFβ1 induced an enhanced mRNA expression of TGFRII-cascade target genes such as Pai-1, Fibronectin, Collagen IV, BMP4, and CTGF (Fig. 1B). Upregulated mRNA levels of TGFβ-receptor subtypes I and III and ligands TGFβ_1, 2, 3_ reflect a highly stabilized activity of the entire TGF-β-system. These effects were reduced or not seen when NVP-13 and NVP-13 + TGFβ1 were added to cell media. Both NVP-13 and NVP-13 + TGFβ1 reduced the phosphorylation of Smad2 and FoxO4 proteins, while only the NVP-13 treatment group also reduced Pai-1 expression (Fig. 1C). Since phosphorylation of Smad2 and FoxO4 is known to inhibit stem cell activity (see below), these *in vitro* data indicate a potentially positive effect of NVP-13 on neurogenesis. This “stem cell favoring scenario” is also pointing in the direction of NVP-13 as a protector of the neurogenic niche underlined by slightly enhanced Msi-1 mRNA expression in the NVP-13 and NVP-13 + TGFβ treatment groups, reduced Msi-1 mRNA expression with TGFβ1 alone, and a significantly increased doublecortin (DCX) expression in the NVP-13-group compared to controls (Fig. 1D, E).

### *In Vivo* Effects of NVP-13 on Target Regulation and Intracellular Pathways: NVP-13 is a Safe and Effective Compound in Cynomolgus Monkeys

**Fig. 2:**
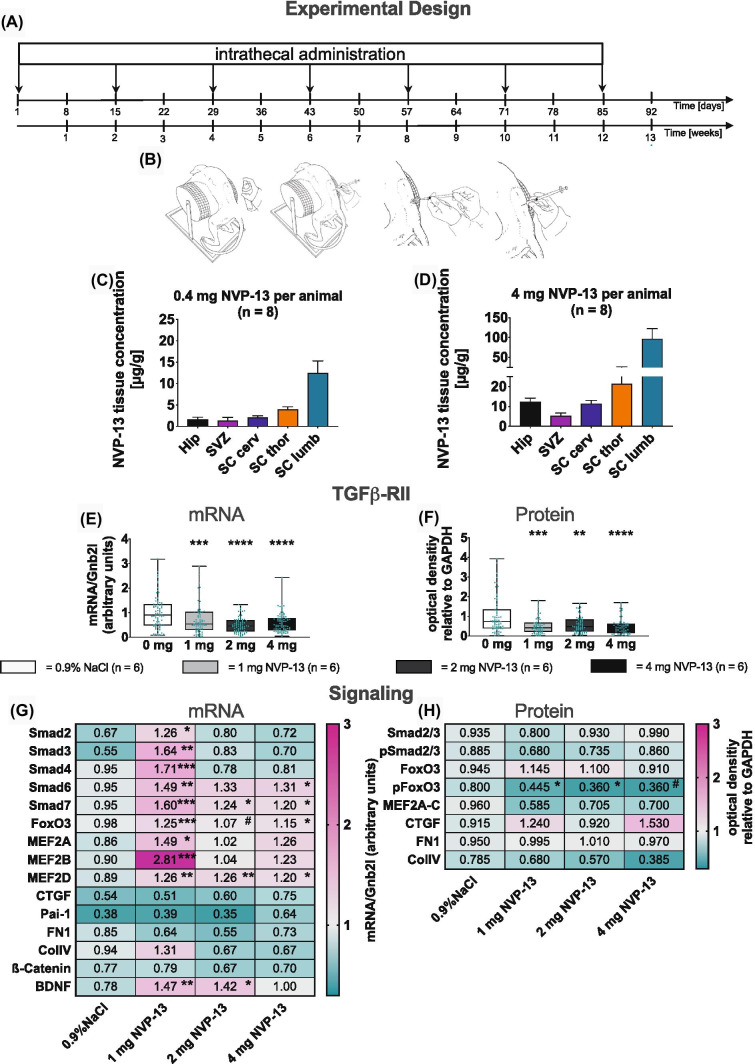
*In vivo* effects of NVP-13 on target regulation and intracellular pathways. 0.9% NaCl (saline control, *n* = 6) and NVP-13 (1 mg, *n* = 6; 2 mg, *n* = 6; 4 mg, *n* = 6) were injected repeatedly over a 13-week paradigm **(A)**. Following disinfection, intrathecal (i.th.) administration was performed via lumbar puncture between L3 and L5 by slow manual bolus infusion over 1 min to anesthetized animals. The needle (with syringe) was left in the dosing site for at least 30 s after aCSF flush. It was documented that CSF flow was present before dosing, that the position of the needle opening was facing towards the head of the animal prior to dose administration, and that the needle (with syringe) was left in dosing site for at least 30 s after aCSF flush **(B)**. In a previous and comparable experiment, NVP-13 was detectable in a dose-dependent manner in CNS regions relevant for neurogenic niche activity **(C**,** D)**. Thirteen weeks of repeated i.th. NVP-13 administrations resulted in significantly reduced TGFβ-RII mRNA and protein expression levels within the spinal cord, SVZ, and hippocampus tissue (areas combined) compared to saline-treated animals **(E**,** F)**. Canonical and non-canonical signaling factors exhibited significantly enhanced mRNA expression levels within CNS tissue following 1 mg NVP-13 administration (Smad2, 3, 4, 6, 7, FoxO3, MEF2A, MEF2B, MEF2D), 2 mg NVP-13 administration (Smad7, MEF2D), and 4 mg NVP-13 administration (Smad6, 7, FoxO3, MEF2D) compared to saline-treated monkeys. Further, mRNA levels of the neurogenesis-mediating trophic factor BDNF were significantly enhanced within CNS tissue following 1 mg and 2 mg NVP-13 treatment **(G)**. Protein phosphorylation of the non-canonical signaling molecules FoxO3 and protein expression of MEF2A-C were significantly enhanced within CNS tissue following 13 weeks of repeated NVP-13 administrations (pFoxO3 1 mg, 2 mg; MEF2A-C 1 mg). Further, there was a tendency towards increased FoxO3 phosphorylation within the 4-mg treatment group compared to saline-treated monkeys **(H)**. All parameters were tested for Gaussian distribution using D’Augostino-Pearson’s omnibus normality test. Afterwards, all parameters were analyzed using a Kruskal-Wallis test followed by Dunn’s post hoc test. Data are presented as mean + standard error of the mean (SEM) **(C**,** D)**, median with min to max **(E**,** F)**, and median **(G**,** H)**. #*p* < 0.1 vs 0.9% NaCl (Trend); *p < 0.05; **p < 0.01; ***p < 0.001; ****p < 0.0001 vs 0.9% NaCl (significant)

For evaluating *in vivo* tolerance of NVP-13 treatment, potential toxicology, and efficacy, male and female cynomolgus monkeys were injected intrathecally (i.th., into the lumbar cerebrospinal fluid) (i) with 0.4 mg and 4 mg NVP-13 (per animal) in a pre-GLP protocol and (ii) with NVP-13 (1 mg, 2 mg, 4 mg) and physiological saline as controls every 2nd week (Fig. 2A, B) in a regulatory 13-week GLP protocol. NVP-13 was very well tolerated, with “No Observed Adverse Effect Level” up to 4 mg/animal. As shown recently [32–35], similar to Nusinersen treatment for SMA (spinal muscular atrophy), NVP-13 showed effective brain concentrations between 2 and 100 μg NVP-13/g tissue, regulating TGFβRII expression in CNS subregions SC, SVZ (subventricular zone), and HIP (hippocampus) (Fig. 2C, D). Here, NVP-13 significantly reduced TGFβRII target expression *in vivo* by nearly 50% (SGZ, SVZ, and SC—all regions/areas are combined) (Fig. 2E, F). Variability within data points is due to the variability of drug concentrations as shown in Fig. 2C, D).

### NVP-13 Downregulates TGFβ Signaling in Cynomolgus Brains

Next, we examined the *in vivo* effects of i.th. NVP-13 treatment in cynomolgus monkey brains on TGFβ downstream signaling, by analyzing canonical and non-canonical pathway effectors. Interestingly, our results indicate the lowest NVP-13-dose (1 mg) in our GLP experiments to exert the most prominent effects on intracellular signaling. On mRNA levels, both canonical and non-canonical pathways displayed a significant regulation at 1 mg dose resulting in downregulation of TGFβ signaling (Fig. 2G). The non-canonical pathways, especially for MAP kinase and PI3K indicated by transcript levels of transcription factors MEF2 and FoxO3, respectively, had a more prominent significant protein downregulation/dephosphorylation than the Smad-dependent pathways (Fig. 2G, H). Enhanced mRNA expression of some factors promoting TGFβ signaling might reflect compensatory mechanisms antagonizing the induced net reduction of TGFβ signaling.

### Upregulated TGFβ signaling Arrests Neurogenesis in Cynomolgus Monkeys, NVP-13 Recovers Neurogenic Niche

**Fig. 3:**
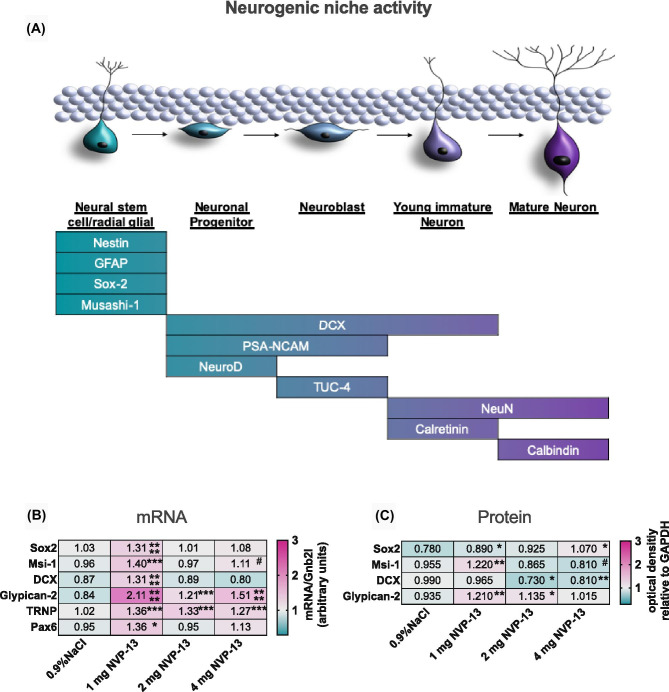
*In vivo* effects of NVP-13 on neurogenic niche markers. Neurogenesis represents a multistep process reflected by distinct and characteristic markers for each single phase and cellular population **(A)**. Following repeated i.th. administrations for 13 weeks (0.9% NaCl; saline control, *n* = 6) and NVP-13 (1 mg, *n* = 6; 2 mg, *n* = 6; 4 mg, *n* = 6), the lowest dose seemed to exert the highest efficacy on activating neurogenic niche activity with significantly enhanced mRNA expression levels of Sox-2, Msi-1, DCX, Glypican-2, TRNP, and Pax6. Further, Glypican-2 and TRNP mRNA levels were also significantly enhanced for the two higher doses **(B)**. Sox-2, Msi-1, and Glypican protein expression levels were significantly enhanced for the 1-mg NVP-13 group compared to saline-treated animals. Sox-2 levels were also significantly increased following administration of 4 mg and glypican-2 following 2 mg NVP-13. DCX protein expression was unchanged following 1 mg NVP-13 administration and significantly decreased after 2 mg and 4 mg repeated NVP-13 administrations compared to saline-treated monkeys **(C)**

As discussed above, several factors indicate that the upregulated endogenous TGFβ-system negatively impacts on neurogenic niche activity *in vivo* [20, 36–39] (Fig. 6). Neurogenesis as a multistep process is characterized by distinct differentiation markers for each development phase and cell population (Fig. 3A). Here, we examined prominent neural stem cell markers Sox-2 and Msi-1 to evaluate NVP-13 efficacy on neurogenesis in the adult neurogenic niche (SGZ, SVZ, and SC—all areas combined). Interestingly, here as well, the lowest NVP-13 dose (1 mg) exerted the highest efficacy in activating neural precursors in the adult neurogenic niche. Sox-2 and Msi-1 mRNA and protein expression levels were significantly enhanced following 13 weeks of 1 mg i.th. NVP-13 application (Fig. 3B, C). The same applied for mRNA levels of the differentiation marker DCX, present in the intermediate phase of adult niche neurogenesis. Notably, NVP-13 administration strongly enhanced mRNA and protein levels of the clinical neurogenesis marker Glypican (Fig. 3B, C).

### TGFβ Signaling and NVP-13 in Downstream Regulation of Cynomolgus Neurogenesis

Analysis of canonical and non-canonical TGFβ-signaling shed some light upon mechanisms forming the basis of the neurogenic niche activity modulation. Significantly enhanced NVP-13-induced Smad7 mRNA levels (most prominent for the 1 mg group, Fig. 2G) may lead to reduced canonical TGFβ-signaling, and thereby increased expression of the neural stem cell markers Sox-2 and Msi-1. This hypothesis was supported by slightly reduced phosphorylation levels of Smad2/3 (Fig. 2H). Enhanced Smad2, 3, 4, 6 mRNA expression might reflect an endogenous mechanism, which compensates for reduced TGFβ signaling (Fig. 2G).

In line with these observations, repeated i.th. NVP-13 administration resulted in significant reduction of suppressive MEF2A-C within CNS tissue (Fig. 2H), here strongest for the 1-mg dose, which is another strong argument that NVP-13 treatment is specifically promoting neurogenic niche activity. In addition, enhanced FoxO3, MEF2A, MEF2B, and MEF2D mRNA expression might reflect compensatory mechanisms competing with reduced TGFβ-R signaling.

To strengthen our hypothesis that repeated NVP-13 administrations will enhance neurogenesis by downregulating preferably non-canonical TGFβ signaling, we investigated the expression levels of the “late” neurogenesis markers DCX and Glypican-2 (Fig. 3B, C). We again found that the lowest dose of NVP-13 exerted the most effective upregulation of these two markers. This was even more pronounced compared to the “early” neural stem cell markers Sox-2 and Msi-1. DCX mRNA levels showed a strong upregulation at the 1-mg NVP-13 dosage but no change for the higher doses whereas protein levels showed significant downregulations for the 2- and 4-mg doses. For glypican, the neurogenic stem cell marker most validated for clinical application, the dose response for mRNA upregulation was significant for all three doses (optimum at 1 mg dose), but here, protein levels were also significantly (at 1 mg and 2 mg doses) upregulated in the same pattern. Aside from gain in evidence for the efficacy of NVP-13, this is a very optimistic perspective to use glypican as a promising clinical biomarker for neurogenesis.

### NVP-13 Treatment Downregulates TGFβ Signaling and Thereby is Accelerating Cynomolgus Neurogenesis

**Fig. 4:**
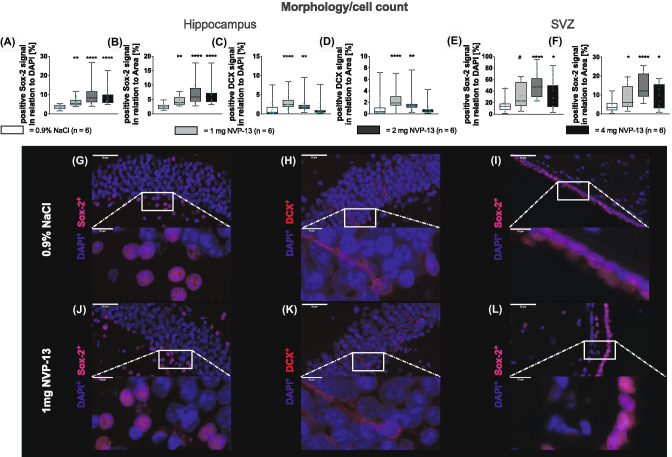
*In vivo* effects of NVP-13 on morphology and cell count. Neural Sox-2-positive stem cells were significantly enhanced within the granular layer of the dentate gyrus of the hippocampus **(A**,** B)** and the ventricular site of the lateral ventricle (**E**, **F**) for all NVP-13 doses. The number of DCX-positive cells was significantly enhanced within the granular layer of the dentate gyrus of the hippocampus **(C**,** D)** following repeated i.th. NVP-13 (1 mg, 2 mg) administrations compared to NaCl-treated animals, whereas for higher dose, the effect was less pronounced. Representative pictures of the positive staining are shown in (**G**–**I)** (0.9% NaCl) and **(J**–**L)** (1 mg NVP-13). All parameters were tested for Gaussian distribution using D’Augostino-Pearson omnibus normality test. Afterwards, all the parameters were analyzed using a Kruskal-Wallis test followed by Dunn’s post hoc test. Data are presented as median **(B**,** C)** and median with min to max **(D**–**I)**. #p < 0.1 vs 0.9% NaCl (Trend); *p < 0.05; **p < 0.01; ***p < 0.001; ****p < 0.0001 vs 0.9% NaCl (significant)

Next, we wanted to merge the signaling data with functional analysis of NVP-13 treatment effects upon adult neurogenic niches, i.e., the SGZ and the SVZ, by immunohistochemistry for Sox-2 and DCX. Numbers of Sox-2-positive cells were significantly enhanced in both areas with increasing NVP-13 dosages (Fig. 4A, B, E, F). DCX-positive cells were significantly enhanced following 1 and 2 mg NVP-13 administrations compared to controls, whereas for higher doses, the effect was less pronounced (Fig. 4C, D). Immunohistochemical images illustrate these findings for SGZ (SOX-2^+^ cells and DCX^+^-cells: Fig. 4G, J, H, K) and for SVZ (SOX2^+^ cells: Fig. 4I, L). Quantifications were done either with respect to DAPI^+^ signal of the area of interest or in a second approach, to the area of analysis itself. Both ways of quantification revealed comparable results (Fig. 4A–F).

### Clinical Relevance of Interfering with TGF$\upbeta$-signaling by NVP-13-Mediated Reduction of TGF$\upbeta$-RII

**Fig. 5:**
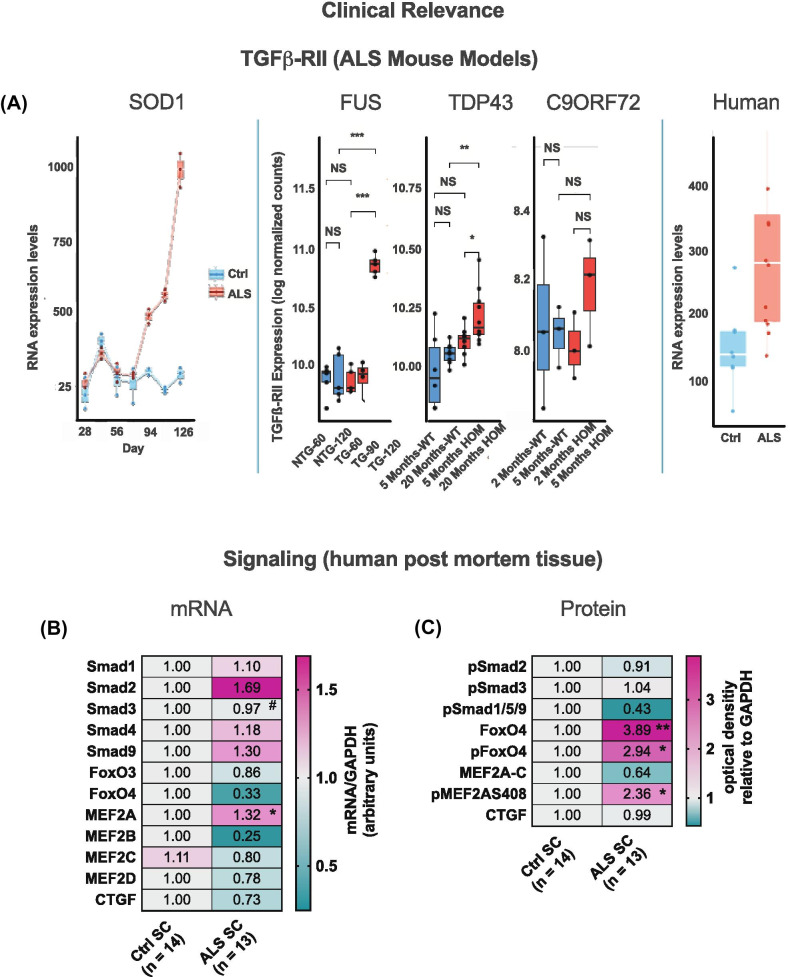
Clinical relevance and proposed mechanisms of NVP-13. **(A)** TGFβ-RII mRNA expression in the timeline of ALS progression in the *SOD1*^*G93A*^, FUS, TDP-43, and C9ORF72 mice (*n* = 3 per genotype and timepoint) (second panel) and sporadic ALS patients (*n* = 12) and controls (*n* = 10) (third panel) **(A)**. **(B**,** C)** Human post-mortem spinal cord tissue of ALS patients (*n* = 13) exhibits significantly enhanced non-canonical TGFβ-mediated signaling with the increased expression and phosphorylation of FoxO4 and MEF2AS408 levels compared to controls (*n* = 14; **B**, **C**). Data were tested for Gaussian distribution using D’Augostino-Pearson’s omnibus normality test. Afterwards, all parameters were analyzed using a two-tailed Student’s *t* test or Mann–Whitney test, depending on Gaussian distribution. Data are presented as median (heat maps)

**Fig. 6:**
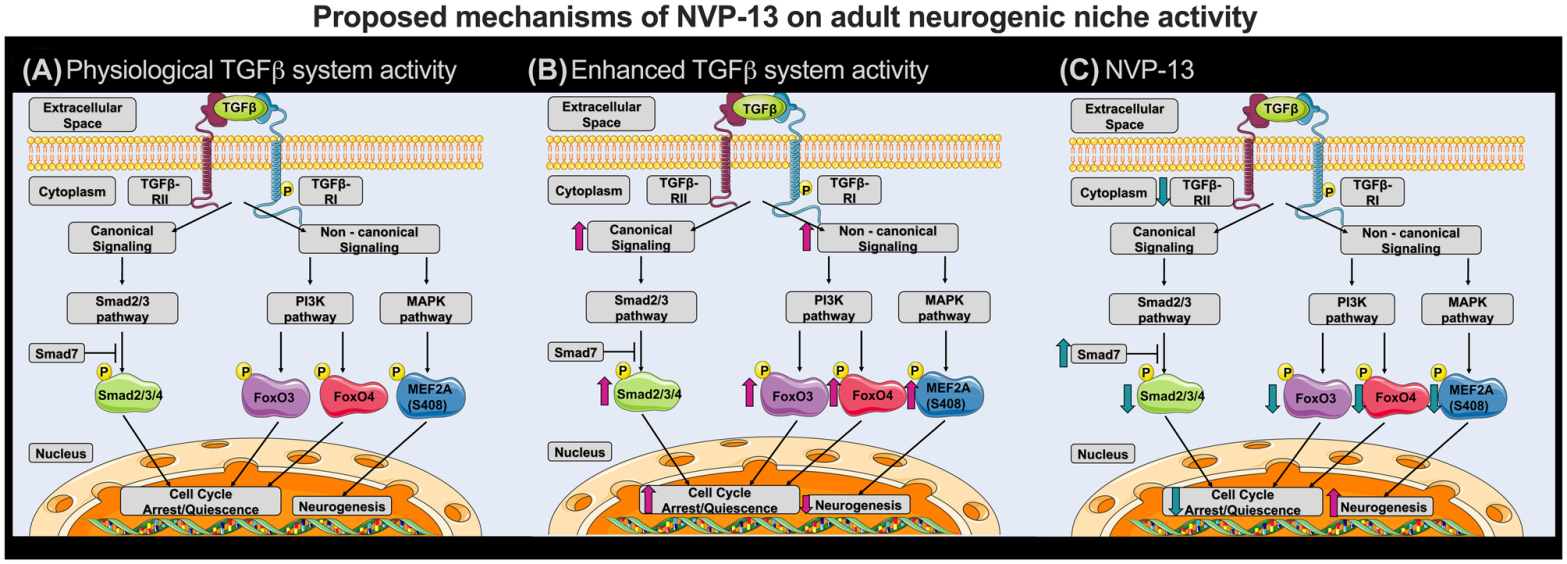
Proposed mechanisms of NVP-13 on neurogenic niche activity repeated i.th. NVP-13 administrations enhanced adult neurogenic niche activity within cynomolgus monkeys by modulation of both, the canonical Smad-dependent signaling cascade and non-canonical Smad-independent pathways. In more detail, NVP-13 reduces stem cell quiescence/cell cycle arrest by inhibiting Smad2/3 signaling via increased Smad7 amounts. Further, stem cell quiescence/cell cycle arrest was reduced by inhibiting the non-canonical PI3K and MAPK pathways with the downstream transcription factors pFoxO3 and MEF2A leading to enhanced neurogenesis **(A**-**C)**. #*p* < 0.1 vs 0.9% NaCl (Trend); *p < 0.05; **p < 0.01; ***p < 0.001; ****p < 0.0001 vs 0.9% NaCl (significant)

Expression analysis of brains and spinal cord from disease models SOD1^G93A^, FUS, TDP-43, and C9ORF72 SC indicated an increased TGFβ-RII mRNA expression in the timeline of ALS progression. TGFβ-RII, where upregulation in the CNS is a constant parameter once symptoms in the animals have manifested and appears therefore to be closely related to clinical disease stage and/or disease progression. In this context, we examined human spinal cord tissue samples from deceased ALS patients using tissue from non-diseased individuals (mostly traffic accidents) as controls and discovered that TGFβ-RII expression was indeed upregulated in ALS patient SC tissue (Fig. 5A). We also identified significantly enhanced phosphorylation of FoxO4 and MEF2A S408 (Fig. 5C), which are elements responsible for TGFβ-RII mediation of neurogenic niche activities via cell cycle modulation (Fig. 6A–C). This clearly indicates that ALS-related neuroinflammation activates FoxO4 and MEF2 proteins in human ALS tissue. Taken together, our monkey data thus show that NVP-13 is reactivating neurogenesis by reducing the activities of these transcription factors.

## Discussion

In the present study, we provide initial evidence that attenuation of upregulated TGFβ signaling prominent for neurodegenerative disorders in general, and ALS in particular, may recondition the adult neurogenic niche and thereby enhance its activity. Our *in vitro* and *in vivo* data as well as analysis of human postmortem SC tissue suggest and confirm an inverse relation between the activity of the TGFβ-system and the status of the neurogenic niche.

*In vitro* NVP-13 treatment could efficiently downregulate target mRNA and target protein alone, or in presence of the ligand TGF-β1, similar to the clinical situation in a neurodegenerative process. We confirmed our biochemical data by immunocytochemistry showing effective downregulation of the target protein TGFβ-RII within neuronal precursor cells, a finding we described earlier in more detail [31]. TGFβ-RII antagonization also reverted upregulated markers for fibrotic scarring and extracellular matrix changes (Fig. 1B, C). Additionally, neurogenic niche activity could be reactivated (Fig. 1D, E) as evidenced by mRNA downregulation of early and late stem cell markers MSI-1 and DCX. NVP-13 alone had a significant stimulatory effect on these markers which, when administered together with TGF-β, was leveraged for DCX and significantly stimulated for the early stem cell marker MSI-1.

We used the pre-GLP and GLP-Tox program to treat healthy non-human primates (to increase specificity and to reduce off-target and off-sequence effects, a top candidate (NVP-13), being effective in humans and non-human primates exclusively, was chosen) with NVP-13 to quantify the effects of antagonizing the TGF system when administered together in a physiological milieu *in vivo*. Here, we are not looking at an animal model for neurodegeneration with upregulated TGF-β_1_. The repeated i.th injections resulted in sufficient drug levels in spinal cord and brain as determined with an NVP-13-specific *in vitro* probe, reaching concentrations between 2μg (0.35 nM) and 100μg (17.0 nM) NVP/g tissue obtained in a very broad therapeutic window (0.4 to 4 mg single dose per monkey) (Fig. 2C, D). Variation of drug levels is mainly due to withdrawal of the anesthetic agent immediately after drug injection, as prescribed by animal welfare protection, which results in almost immediate normal physical activities of the monkeys. Human patients, however, would rest in a supine position for 2 h after i.th. injection, which would lead to more reliable and homogeneous drug concentrations in the CNS.

Injection of NVP-13 into cerebrospinal fluid of Cynomolgus monkeys resulted in a robust downregulation of TGFβ-RII mRNA and protein (Fig. 2E, F) in all areas measured (SC—lumbar, thoracic, and cervical levels, SGZ, SVZ). Although neurogenesis is normal in healthy young non-human primates, the repeat i.th. injection of NVP-13 resulted in a significant and dose-related increase in numbers of newborn neurons that are characterized by SOX2+ as a marker for early progenitor cells (Fig. 3A). We were able to show this increase in cell numbers for the subgranular zone of the dentate gyrus (SGZ) as well as for the subventricular zone (SVZ) (Fig. 4A–F). To look at later stages of neuronal stem cell differentiation, we used the neuronal marker DCX for neurogenesis and found a significant increase in DCX-positive cells. The increase in SOX2+ and DCX+ cells was dose dependent, with the strongest effects between 1 and 2 mg NVP-13 dosages. Further data points on pharmacokinetics and pharmacodynamics (PK and PD) will be needed within the initial clinical evaluation with respect to application in resting humans on a supine position. Despite these limitations, the interesting findings remain that we are able to substantially elevate neurogenesis in these healthy young animals as a potential therapeutic approach—without any toxicity of NVP-13. The rapid increase in neurogenesis within 13 weeks of therapy also creates a measure of optimism about the time course in future clinical applications. However, these data will have to be verified for human patients and need much more detailed specification.

Analysis of canonical and non-canonical TGFβ signaling shed some light on mechanisms forming the basis of these reported modulations (see Fig. 6A–C). The canonical pathway stimulates cell cycle arrest and quiescence via its effector *pSmad2/3/4-complex*, whereas Smad7 inhibits Smad-mediated TGFβ signaling. Thus, significantly elevated NVP-13-mediated Smad7-mRNA expression levels (most prominent for the 1 mg group, Fig. 2G) may lead to reduced canonical TGFβ signaling and thereby promote neurogenic niche activity. This hypothesis is supported by slightly yet not significant, reduced phosphorylation levels of Smad2/3 (Fig. 2H).

Our data indicate also that *non-canonical pathways*, such as the *PI3K* and *MAPK* pathways, are critically involved in modulating neurogenic niche activity. The PI3K pathway is a major regulator of cell cycle activity involving the transcription factors FoxO3 and FoxO4. Following activation, phosphorylated FoxO3 and FoxO4 (pFoxO) translocates to the nucleus and enforces cell cycle arrest by blocking S-phase progression and cell cycle arrest in G1/S and G0/G1 phases [40]. Therefore, the NVP-13-induced reduction of pFoxO3 (Fig. 2H) might dissolve the blockade of cell cycle arrest/quiescence, and thereby promote neurogenic niche activity. The relevance of these mechanisms to be potentially very effective in ALS patients is underlined by the significantly upregulated protein levels of FoxO4/pFoxO4 found in the human postmortem analysis (also compared to normal control SC: Fig. 5C).

The MAPK pathway represents another powerful candidate for modulating non-canonical TGFβ-RII signaling–mediated neurogenic niche activity. MEF2A, a transcription factor being phosphorylated at S408 downstream of MAPK activation [41], negatively controls transcription of genes regulating cell cyclus, proliferation, neuronal morphology, and connectivity [41]; its activity has been associated with reduced synapse development [42], neurite retraction [41], and neurogenesis and promoting cell death and fibrosis [42], which all play important roles in neurodegeneration. MEF2A mRNA expression and protein phosphorylation are significantly upregulated in ALS autopsy material, but protein expression is low (due to degradation?) (Fig. 5B, C). NVP-13 treatment with TGF-β1 present downregulates MEF2A expression *in vitro* (mRNA and protein: Fig. 1B, C). However, NVP-13 administration alone enhances MEF2A mRNA expression but results in low protein levels. This shows that the NVP-13 treatment schedule as demonstrated here is able to enhance mRNA but not protein expression, there is quite some activity in this system upon treatment, which may be due to the short period of therapy, and its final evaluation comes with the caveat that healthy monkeys were treated who did not show TGFβ dysregulation.

At first, the dose-dependent activity profile of NVP-13 with the lowest dose exerting the most prominent effects was unexpected. A high NVP-13 dose might be able to lead neural stem cells towards a glial cell fate and undergo gliogenesis. This is unlikely, however, as astrocyte and oligodendrocyte markers GFAP and S100β, and Olig2, respectively, were unaltered at 4 mg NVP-13 (Supplementary Fig. 3). Another explanation may be that excessive inhibition of TGFβ signaling impairs neural maturation [39], but we have no data yet to substantiate this and will need to look at the mechanisms more carefully, which is planned for a chronic GLP-Toxicity study. We want to emphasize again that this study was performed in young and healthy monkeys with a juvenile, yet unaltered, TGFβ-system, and not in a TGFβ-upregulated diseased condition.

Animal and human studies indicate TGFβ-system dysregulations and impaired neurogenesis as a common feature of neurodegenerative disorders, particularly in ALS, Alzheimer’s disease, and Huntington’s disease [14, 43–47]. A very recent review by Kandasamy et al. describes the role of TGFβ in the context of vascular dementia and highlights the TGFβ-system as a therapeutic target to reinstall regenerative plasticity [48]. An *in vivo* mouse study recently demonstrated an accelerated disease progression in SOD1^G93A^ mice driven by an astrocyte-specific overproduction of TGFβ1 [49, 50]. This led to inhibition of microglia activation and thereby to a reduced neuroprotective milieu. Importantly, intraperitoneal administration of a TGFβ signaling inhibitor dampened disease progression and extended survival of SOD1^G93A^ mice. The significantly enhanced TGFβ-RII mRNA levels in SOD1G93A, FUS, TDP-43, and C9ORF72 mice, and mechanistic insights from ALS *in vivo* animal models as well as post mortem tissue analysis from ALS patients [14] (Fig. 5A), all strengthen our hypothesis: NVP-13 treatment is able to ameliorate disease progression and induce potential repair mechanisms.

Therapeutic interference with NVP-13 is accompanied by only a very mild inflammatory response within the target tissue, making NVP-13 treatment a very safe intervention (Supplementary Fig. 2) [51]. Future clinical trials should include a comprehensive monitoring of the accompanying inflammatory responses. Taken together, our approach aims to influence the TGFβ-system in the neurogenic niche, including classical TGF-β target areas such as ALS milieu factors (fibrosis, autophagy, immune dysfunction), to enhance neurogenesis and repair of damaged neurons. Downregulation of an overactive TGF-β-system with excellent tolerance will be a highly attractive approach to treat neurodegeneration efficiently. Not only stopping the disease would be a real chance but also the perspective of organ repair. However, our approach is now in need of clinical confirmation.

Naturally, this study has a number of serious limitations. The main drawback is construed in the nature of the test compound NVP-13, in that it only addresses the human and non-human primate mRNA sequence of the target. This was intended with the purpose of reducing potential clinical toxicity induced by off-sequence and off-target effects, but our NVP-13 compound can thus not be employed in current ALS animal models [31]. Another limitation is the inability to investigate the same areas of pathology in human patients with ALS, normal tissue controls, and in the monkeys, i.e., brain or spinal cord, which is an enormous challenge. This will hopefully be overcome with a thorough biomarker (blood, saliva, CSF) program as molecular surrogate markers in the clinical development phase, where different aspects may be reflected by specific biomarker tools. Finally, within a GLP-Tox study, the regulatory study program issues are dominant, which limit morphological analysis in specific areas like the spinal cord, due to the actual time courses of autopsies. For this reason, we did not have unlimited tissue available and could only obtain spinal cord drug levels and determine mRNA and protein concentrations for the target TGFβ-RII, but not perform extensive immunocytochemistry.

## Material and Methods

### Antisense Oligonucleotide (NVP-13) Characteristics

NVP-13, a gapmer LNA-Antisense Oligonucleotide, was designed as the drug candidate to specifically hybridize with the mRNA for TGFβ-RII. To increase specificity and to reduce off-target and off-sequence effects, a top candidate (NVP-13), being effective in humans and non-human primates exclusively, was chosen from several tested gapmers.

### *In Vitro* Experimental Design

Human neural progenitor cells (ReNcell CX® cells, no. SCC007, Merckmillipore; not listed in the database of commonly misidentified cell lines, cell lines have not been authenticated) were cultured according to manufacturer’s recommendations. Cells were pretreated with TGFβ1 (50 ng/ml) to simulated diseased conditions. Following 4 days of pretreatment, TGFβ1-containing medium was removed, and cells were cultured in fresh medium containing either PBS (control), NVP-13 (10 µM), TGFβ1 (50 ng/ml), or a combination of NVP-13 (10 µM) and TGFβ1 (50 ng/ml). This procedure was repeated on experimental day 8. On experimental day 12, the medium was removed, and cells were harvested for mRNA, protein, and immunofluorescence analysis. Cell lines were tested regularly (every 4th splitting process) for mycoplasma contamination using a PCR Mycoplasma Kit (PromoCell, no. PK-CA91-3025A). In the case of any positive mycoplasma contamination, cells were discarded.

### *In Vivo* Experimental Design

A 0.9% NaCl (control group) and NVP-13 (three different doses) were injected repeatedly over a 13-week approach. Physical/neurological parameters (general sensomotory aspects, cerebral reflexes (pupillary, orbicularis oculi) and spinal reflexes (patellar, anal) and foot grip reflex, as well as abdominal palpation, body temperature, heart (functional), and lung (functional) auscultation) were investigated directly and following 4 h after administration. If neurological abnormalities were present 4 h after dosing, additional neurological examination time points (in daily intervals) were performed to assess their reversibility/progression. For clinical chemistry, bioanalytics, biomarker determination, and target regulation, blood and CSF samples were taken before every dosing, and tissue samples (liver, kidney, spinal cord, brain) were collected in the end of the study. No animal exclusions were determined. No blinding to group allocation was done.

### Pre-study Procedures

Male (*n* = 3) and female (*n* = 3) cynomolgus monkeys (Macaca fascicularis, Asian (purpose bred)), with the age of 2 to 6 years, weighing 2.2 to 5 kg, were housed in groups (for non-human primate preclinical/safety assessment studies, the guideline-specific animal number is 3 animals per group and sex). Animals were randomized to ensure equal bodyweight per group and sex. All animals were kept under standard laboratory conditions in a climate-controlled room with a minimum of 8 air changes/h (12-h light/dark cycle, 19 to 25 °C, 40 to 70% humidity). All animals received a certified lab diet for primates (LabDiet 5048) twice daily, supplemented by fresh fruits and vegetables and had access to tap water (H_2_O) ad libitum*.* Immediately after each handling/manipulation, the animals received a tasty reward. All experimental procedures are in compliance with the German Animal Welfare Act and are approved by the local IACUC. The non-human primate study was conducted following approval by the LANUV (Landesamt für Natur, Umwelt und Verbraucherschutz, NRW) and registered following file number 84-02.04.2017.A093. This study was performed in compliance with the following guidelines or recommendations:

- European Directive 2001/83/EC and all subsequent amendments
- International Conference on Harmonization (ICH) guideline: guidance on non-clinical safety studies for the conduct of human clinical trials and marketing authorization for pharmaceuticals, M3(R2), issued in EMA as CPMP/ICH/286/95.
- ICH-S3A, Toxicokinetics: a guidance for assessing systemic exposure in toxicology studies, issued in EMA as CPMP/ICH/384/95
- ICH-S4, duration of chronic toxicity testing in animals (rodent and non-rodent toxicity testing), issued in EMA as CPMP/ICH/300/95
- ICH-S6, preclinical safety evaluation of biotechnology-derived pharmaceuticals, issued in EMA as CPMP/ICH/302/95, and first revision, issued in EMA as CHMP/ICH/731,268/1998
- Guideline on repeated dose toxicity, issued in EMA as CPMP/SWP/1042/99 Rev 1

And in the German Drug Law “Arzneimittelgesetz.” All efforts were made to minimize the number of animals used and their suffering. Therefore, the experiment was performed once.

### Dosing Procedure

Prior to dosing vehicle or test item formulation (0.75 mL followed by 0.25 mL of an aCSF (artificial cerebral spinal fluid; aCSF, from R AND D SYSTEMS INC, USA)), a volume ≤ 1.0 mL of CSF was withdrawn for each injection. In order to reduce the risk of contamination, a microincision of the skin was conducted using a 20G needle, before introduction of the spinal needle. Dosing was elaborated with a Pencan Paed® pencil-point needle (25 G, B. Braun Melsungen AG, Germany) to minimize puncture size during the *dura mater* penetration. I.th administration was performed via lumbar puncture between L3 and L5 by slow manual bolus infusion over 1 min to anesthetized animals.

In more detail, the needle (with syringe) was left in dosing site for at least 30 s after aCSF flush. It was documented that (1) CSF flow was present before dosing, (2) that the position of the needle opening was facing towards the head of the animal prior to dose administration, and that (3) the needle (with syringe) was left in dosing site for at least 30 s after aCSF flush. Following the administration, (4) the animal was carefully returned in a transport box and placed in a lying position for 15 min before the antidote was applied. Bepanthen® aseptic wound ointment (contains chlorhexidine and dexpanthenol) was applied immediately after each dosing. Following dosing physical (abdominal palpation, body temperature, heart (functionally) and lung (functionally) auscultation and neurologic (general sensomotory aspects, cerebral reflexes (pupillary, orbicularis occuli) and spinal reflexes (patellar, anal) and foot grip reflex) examinations were conducted 4 h post-dose and respectively daily intervals (if findings were absent), to compare reflex changes (if any) with pre-dose (before sedation) results.

### Standard in Life Parameters

Standard in life parameters comprising physical and neurological examinations, neurobehavioral observations, ophthalmic examinations, cardiovascular investigations, respiratory rate, and clinical pathology was investigated directly and following 4 h after administration. If neurological abnormalities were present 4 h after dosing, additional neurological examination time points (in daily intervals) were performed to assess their reversibility/progression (For details, see respective study plans).

### End of in Life Phase

At the end of in life phases, animals were sedated by an intramuscular ketamine hydrochloride injection followed by an intravenous sodium pentobarbitone overdose prior to exsanguination. For histology, pathology, toxicokinetics, mRNA, and potential biomarker analysis of the brain, spinal cord, liver, kidney, and as much as possible the lung, spleen, and thymus, lymph nodes (mesenteric, mandibular, and inguinal) were collected and preserved accordingly (for details, see respective study plans).

### Human Tissue

Human post-mortem cryopreserved and paraffin-embedded tissues from the spinal cord (SC), the motorcortex (MC), and the occipital lobe (OL) as an internal control were obtained from the German Motor Neuron Disease Network (MND-Network, Albert Ludolph, Ulm), from female and male ALS patients (*n* = 18) and female and male healthy controls (*n* = 17) (characteristics, see Supplementary Fig. 5). We included the motor cortex in the analysis of the current study since the characteristic of ALS is the degeneration of the upper and lower motor neurons. The analysis of spinal cord lysates covered the investigation of the surrounding milieu of the lower motor neurons since they originate within the spinal cord and project to the muscles. For the upper motor neurons, their origin is within the motor cortex. Therefore, we investigated whether these systems are involved or might promote ALS disease progression; we included the motor cortex for the upper motor neuron. For the occipital lobe, it is known that this area is relatively unaffected by ALS and therefore not involved in the immediate disease progression. Due to this, we included this area in the analysis as an internal control, to survey if alterations seen in the spinal cord and motor cortex are specific. Human tissue was obtained according to different ethical votes from the ethics committee at the University of Regensburg (ethics approval 15-101-0053) the MND-network votes from the ethics committee at the University of Ulm (ethics approval: 19/12-2012) and the ethics committee at the University of Regensburg (ethics approval 13-103-0056). Autopsies were explicitly part of the written informed consent for all patients, being seen in the context of the MND-Net. Analytes were measured in technical triplicates for one time.

### qRT-PCR

For mRNA analysis, harvested cell pellets (*in vitro*) and 30 mg of the respective tissue (*in vivo*, human tissue) were taken for RNA isolation using the innuPREP RNA Mini Kit (Analytik Jena, Jena, Germany). Following DNAase digestion (400 ng RNA per 20 µl) using innuPREP DNAase kit (Analytic Jena, Jena, Germany) according to the manufacturer’s recommendations and determination of RNA content (100 ng RNA per 20 µl), the RNA was reversely transcribed into first-strand cDNA with iScript cDNA Synthesis Kit (BioRad, Hercules, USA) according to manufacturer’s recommendations. For mRNA analysis, qRT-PCR was performed using a CFX96 Touch Real-Time PCR Detection System (BioRad, Hercules, USA). All primer pairs were ready-to-use standardized and were mixed with the respective ready-to-use Mastermix solution (Sso Advanced Universal SYBR Green Supermix (BioRad, Hercules, USA)) according to the manufacturer’s instructions (BioRad Prime PCR Quick Guide). As template, 0.25 μl of respective cDNA was used. H_2_O was used as a negative control for qRT-PCR. For relative quantification, housekeeping gene GAPDH (*in vitro*) or Gnb2l (*in vivo*) was used. Afterwards, BioRad CFX Manager 3.1 was used to quantify mRNA-level relative to GAPDH/Gnb2l mRNA. Analytes were measured in technical triplicates for three times.

### Western Blotting

For protein analysis, harvested cells were lysed using M-PER® Mammalian Protein Extraction Reagent (Thermo Scientific, Braunschweig, Germany), and about 30 mg of the respective tissue was lysed using T-PER® Tissue Protein Extraction Reagent (Thermo Scientific, Braunschweig, Germany) containing protease inhibitor cocktail plus (1:100; Carl Roth, Karlsruhe, Germany) and phosphatase inhibitor cocktail 2 (1:100; Sigma Aldrich, St. Louis, USA) according to manufacture instructions. Afterwards, protein concentrations were determined using Pierce Coomassie Plus Assay Kit (Life Technologies). SDS-acrylamide gels (percentage depending on protein size) were produced using TGX Stain Free™ Fast Cast™ Acrylamid Kit (BioRad, Hercules, USA) according to manufactory instructions. Protein samples (1 µg/µl in/M-PER/T-PER; 18 µl) were diluted 1:4 with Lämmli-buffer (6 µl, Roti®-Load1, Roth, Karlsruhe, Germany), incubated at 60°C for 30 min and loaded on the gel with the entire volume of the protein solution. Separation of proteins was performed by electrophoresis using Power Pac Basic Power Supply (BioRad, Hercules, USA) and Mini Protean Tetra cell electrophoresis chamber (BioRad, Hercules, USA) (200 V, 45 min). Following electrophoresis, the proteins were blotted using Trans-Blot Turbo Transfer System (BioRad, Hercules, USA). All materials for western blotting were included in Trans Blot Turbo RTA PVDF-Midi Kit (BioRad, Hercules, USA). The PVDF-membrane for blotting procedures was activated in methanol (Merck Darmstadt, Germany) and equilibrated in 1× transfer buffer. Following blotting (25 V, 1 A, 30 min), membranes were washed (3×, 10 min, RT) with 1× TBS (Roth, Karlsruhe, Germany) containing 0.05% Tween-20 (Roth, Karlsruhe, Germany). Afterwards, the membranes were blocked with 5% BSA (Albumin-IgG-free, Roth, Karlsruhe, Germany), diluted with TBS-T for 1 h at RT; the primary antibodies (diluted in 0.5% BSA in TBS-T; described in Supplementary Table 1) were added and incubated at 4 °C overnight. Next, membranes were washed in TBS-T (3× 10 min, RT) and incubated with the secondary antibody (Anti-rabbit IgG, HRP-linked, 1:10.000, Cell Signaling CS7074S or Anti-mouse IgG, HRP-linked, 1:7.500, Cell Signaling CS no. 7076S; 1 h, RT). Following incubation, blots were washed with TBS-T, emerged using Luminata^TM^Forte Western HRP Substrate (Millipore, Billerica, USA), and bands were detected with a luminescent image analyzer (ImageQuant LAS 4000, GE Healthcare, Munich, Germany). Afterwards, the blots were washed in TBS-T (3 × 10 min, RT) and blocked with 5% milk powder diluted in TBS-T (1 h, RT). For housekeeper comparison, the membranes were incubated with HRP-conjugated anti GAPDH (1:2000 in 0.5% milk powder, Cell Signaling CS no. 8884S, 4 °C, overnight). On the following day, blots were emerged using Luminata^TM^Forte Western HRP Substrate (Millipore, Billerica, USA), and bands were detected with a luminescent image analyzer (ImageQuant LAS 4000, GE Healthcare, Munich, Germany). Finally, the blots were washed with TBS-T (3×, 10 min, RT) and stained using 1 × Roti Blue solution (Roth, Karlsruhe, Germany) and dried at RT. Blots were analyzed using Image Studio Lite Software (Licor, Nebraska, USA). Analytes were measured in technical triplicates for three times. For detailed antibody information (dilutions, company, order number), see Supplementary Table 1.

### Immunofluorescence

For immunocytochemistry, cells were treated and harvested as described before. Following fixation of cells with Roti-Histofix 4% (Roth no. P087.4) (6 min, RT), cells were washed three times with PBS. After blocking cells for 1 h at RT with Blocking Solution (Zytomed no. ZUC007-100), cells were incubated with TGFβ-RII primary antibody (1: 50) over night at 4°C. Afterwards, cell culture slides were washed three times with PBS following incubation with secondary antibody for 1 h at RT). All antibody dilutions were prepared with antibody diluent (Zytomed).

For immunofluorescence, paraffin-embedded SVZ and hippocampus brain sections and frozen spinal cord (lumbar, thoracic, cervical) tissue were cut into 10-µm sections. Paraffin sections were deparaffinized by heating (1 h, 50 °C) followed by a descending EtOH row (2× xylene 5 min each, 100% EtOH and Xylene 1:1, 2 × 100% EtOH, 95% EtOH, 70% EtOH, 50% EtOH 3 min each). Afterwards, antigen retrieval was performed by heating the sections in 10 mM citrate buffer (20 min, 95 °C). Next, the sections were washed with TBS (1× 5 min), and cryosections were fixed in ice-cold PFA (4%, 5 min). In the next step, the slices were washed with TBS-T (3× 5 min, RT) and blocked with a solution composed of TBS, 1% BSA, 0.1% Triton X-100, and 0.2% teleostean gelatin (2 h, RT). The sections were incubated with primary antibodies diluted in blocking solution over night at 4 °C. On the following day, slices were washed with TBS-T (3× 5 min, RT) and incubated with the secondary antibodies diluted in the blocking solution for 1 h at RT. Then, slides were incubated with 1% Sudan Black b solution to reduce lipofuscin-dependent autofluorescence (10 min RT), washed with TBS-T (3 × 5 min), and mounted using ProLong™ Diamond Antifade Mountant with DAPI (Life Technologies). Respective isotype controls and slices only treated with secondary antibodies were used in the approach to verify specificity. Immunofluorescence was detected and visualized using Olympus slide scanner (Olympus VS120). Three 10-µm cross sections from paraffin-embedded CNS sections (taken 100 µm apart) were used for repetitive analysis. For detailed antibody information (dilutions, company, order number), see Supplementary Table 1.

### Electrochemoluminescence

To analyze TGF-β1, 2, and 3 ligand as well as cytokine expression, about 30 mg of lumbar spinal cord tissue (highest NVP-13 concentrations) was lysed using T-PER® Tissue Protein Extraction Reagent (Thermo Scientific, Braunschweig, Germany) according to manufacturer instructions. Afterwards, protein concentration was determined using Pierce Coomassie Plus Assay Kit (Life Technologies), and the final concentration was adjusted to 1 µg/µl. For electrochemoluminescence (Mesoscale Discovery, Maryland, USA), 25 µl of the protein supernatants was used. Tissue expression of TGF-β ligands and cytokines/chemokines was measured in technical triplicates for one time using non-human primate TGF-β Combo U-Plex Assay and non-human Biomarker Group 1 U-Plex Assay Kit. The assay procedures were performed according to manufacturer’s instructions.

### Database Analysis

The mouse *SOD1*^*G93A*^ spinal cord dataset [52] was downloaded from GEO (accession GSE18597). The transcriptomes from mouse models based on mutations in *FUS (truncated 1–359), TARDBP* (mutation Q331K, reference: PMID: 29556029) and *C9Orf72* (GFP-PR28, reference: PMID: 31266945) were downloaded respectively from the following resources: GSE112629, GSE99354, and GSE132108. Raw cell files were obtained and loaded into R using the affy package [53]. Probe annotations and mapping to HGNC symbols were done using the biomaRt R package [54]. Differential expression analysis was performed using the limma package [55]. Downloaded count data were then normalized by using DEseq2 package (v1.12.4) accounting for variables such as genotype, age, tissue, and disease status, depending on the study.The human spinal cord dataset [56] was downloaded from GEO (accession GSE18920). The data from enriched motor neurons was dropped, and only anterior horn samples were kept. Differential expression analysis was again performed using the limma package [55] controlling for gender.

### Statistics

For graph design and statistical comparison, GraphPad Prism 8 was employed. All *in vitro* parameters were tested for Gaussian distribution using the Shapiro-Wilk normality test (samples sizes too small for D’Augostino-Pearson’s omnibus normality test). Afterwards, all parameters were analyzed using a one-way ANOVA followed by Tukey’s post hoc test or a Kruskal–Wallis test followed by Dunn’s post hoc test, depending on Gaussian distribution. All *in vivo* parameters were tested for Gaussian distribution using D´Augostino-Pearson omnibus normality test. Human data were tested for Gaussian distribution using D´Augostino-Pearson omnibus normality test. Afterwards, all parameters were analyzed using a two-tailed Student’s *t* test or the Mann–Whitney test, depending on Gaussian distribution. Data are presented as median (head maps *in vitro*/*in vivo* data), median with min to max (box plots), mean (head maps human data), and mean + SEM (bar charts). Significance was taken at *p* ≤ 0.05, and a trend was noted at *p* ≤ 0.1.

## Supplementary Information

Below is the link to the electronic supplementary material.

Supplementary file1 (PDF 1255 KB)

Supplementary file2 (PDF 1255 KB)

Supplementary file3 (PDF 1255 KB)

Supplementary file4 (PDF 1255 KB)

Supplementary file5 (PDF 1255 KB)

Supplementary file6 (PDF 1255 KB)

Supplementary file7 (PDF 1255 KB)

Supplementary file8 (PDF 1255 KB)

Supplementary file9 (PDF 1255 KB)

Supplementary file10 (PDF 1255 KB)

Supplementary file11 (PDF 1255 KB)

Supplementary file12 (PDF 1255 KB)

Supplementary file13 (PDF 1255 KB)

Supplementary file14 (PDF 1255 KB)

Supplementary file15 (PDF 1255 KB)

Supplementary file16 (PDF 1255 KB)

Supplementary file17 (PDF 1255 KB)

Supplementary file18 (PDF 1255 KB)

Supplementary file19 (PDF 1255 KB)

Supplementary file20 (PDF 1255 KB)

Supplementary file21 (PDF 1255 KB)

Supplementary file22 (PDF 1255 KB)

Supplementary file23 (PDF 7609 KB)

Supplementary file24 (PDF 7604 KB)

Supplementary file25 (PDF 492 KB)

Supplementary file26 (PDF 445 KB)

Supplementary file27 (PDF 331 KB)

Supplementary file28 (PDF 3419 KB)

Supplementary file29 (PDF 529 KB)

## Data Availability

The authors declare that (the/all other) data supporting the findings of this study are available within the paper (and its supplementary information files). Additional information/data on NVP-13 that support the findings of this study are available from the corresponding author upon reasonable request.
