# Supplementary material for: Reconditioning the Neurogenic Niche of Adult Non-human Primates by Antisense Oligonucleotide-Mediated Attenuation of TGFβ Signaling
Source: Neurotherapeutics. 2021 Apr 15;18(3):1963–79. doi: 10.1007/s13311-021-01045-2 (PMC8609055; doi:10.1007/s13311-021-01045-2)
Supplement: Supplementary file 23 — Supplementary file23 (PDF 7609 KB) [file 13311_2021_1045_MOESM23_ESM.pdf]

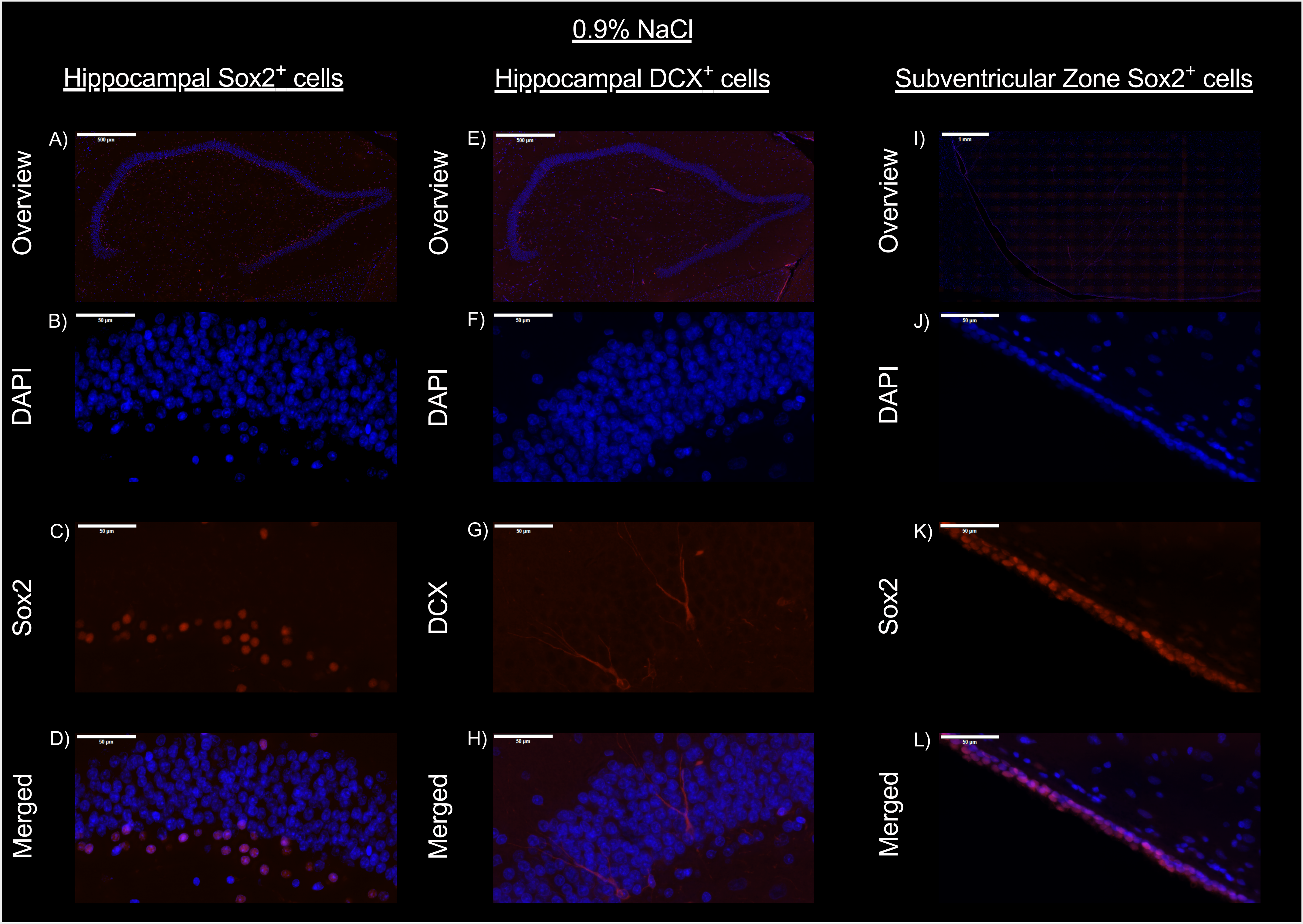

Peters et al., Supplementary Figure 1
