# Supplementary figures and images for: Reconditioning the Neurogenic Niche of Adult Non-human Primates by Antisense Oligonucleotide-Mediated Attenuation of TGFβ Signaling
Source: Neurotherapeutics. 2021 Apr 15;18(3):1963–79. doi: 10.1007/s13311-021-01045-2 (PMC8609055; doi:10.1007/s13311-021-01045-2)

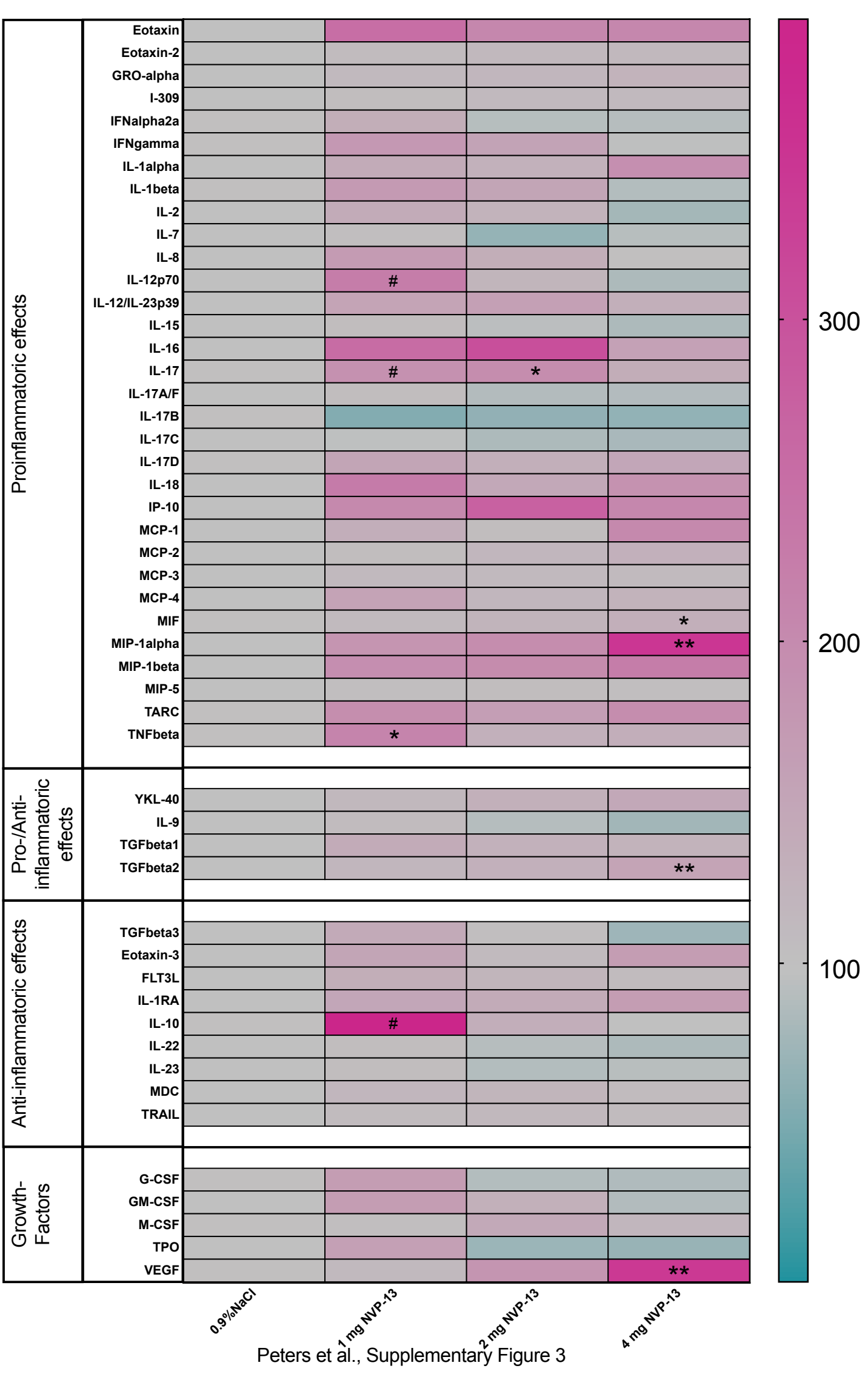

Supplement: Supplementary file 25 — Supplementary file25 (PDF 492 KB) [file 13311_2021_1045_MOESM25_ESM.pdf]

# CNS markers

## mRNA

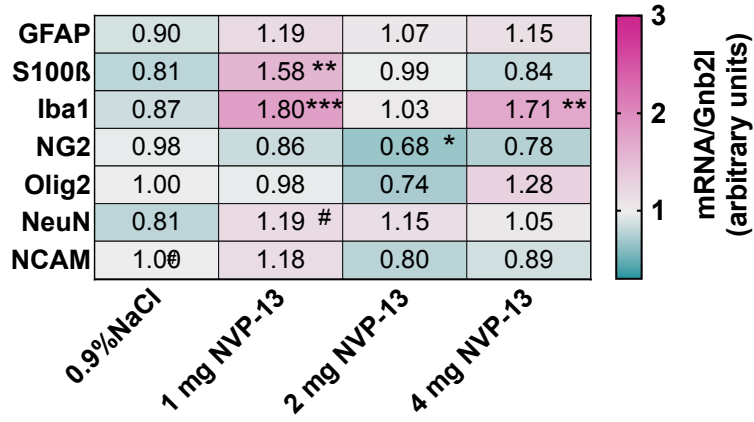

## Protein

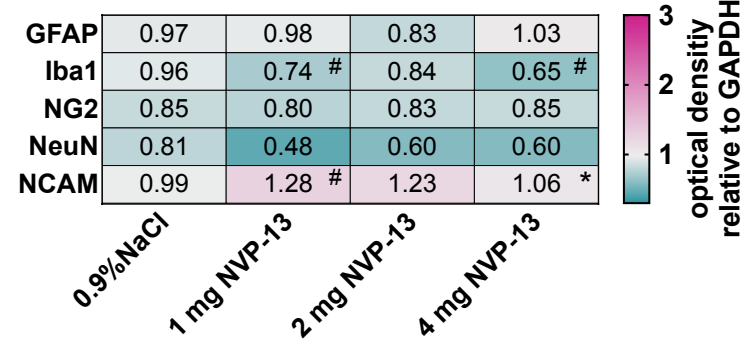

Supplement: Supplementary file 26 — Supplementary file26 (PDF 445 KB) [file 13311_2021_1045_MOESM26_ESM.pdf]
