# Supplementary material for: Reconditioning the Neurogenic Niche of Adult Non-human Primates by Antisense Oligonucleotide-Mediated Attenuation of TGFβ Signaling
Source: Neurotherapeutics. 2021 Apr 15;18(3):1963–79. doi: 10.1007/s13311-021-01045-2 (PMC8609055; doi:10.1007/s13311-021-01045-2)
Supplement: Supplementary file 27 — Supplementary file27 (PDF 331 KB) [file 13311_2021_1045_MOESM27_ESM.pdf]

		Patient No.	Age	Sex	Diagnosis	Spinal Cord	Motor Cortex	Occipital Lobe
Post Mortem CNS tissue	Controls	1	43	male	non-ALS	yes	yes	yes
		2	61	male	non-ALS	yes	yes	yes
		3	66	male	non-ALS	yes	yes	yes
		4	64	male	non-ALS	yes	yes	yes
		5	35	male	non-ALS	yes	yes	yes
		6	59	male	non-ALS	yes	yes	yes
		7	67	female	non-ALS	yes	yes	yes
		8	75	female	non-ALS	yes	yes	yes
		9	74	male	non-ALS	yes	yes	yes
		10	53	female	non-ALS	yes	yes	yes
		11	44	male	non-ALS	yes	no	no
		12	71	female	non-ALS	yes	no	no
		13	62	male	non-ALS	yes	no	no
		14	84	female	non-ALS	yes	no	no
		15	70	male	non-ALS	yes	no	no
		16	78	female	non-ALS	no	yes	no
		17	42	female	non-ALS	no	yes	no
	ALS Patients	1	61	male	ALS	yes	yes	no
		2	75	male	ALS	no	yes	no
		3	66	female	ALS	yes	yes	yes
		4	61	male	ALS	yes	yes	yes
		5	75	female	ALS	no	yes	yes
		6	75	male	ALS	yes	yes	yes
		7	76	female	ALS	yes	yes	no
		8	75	male	ALS	yes	yes	yes
		9	43	male	ALS	no	yes	yes
		10	61	female	ALS	yes	yes	yes
		11	54	female	ALS	yes	yes	no
		12	18	male	ALS	yes	yes	no
		13	60	female	ALS	yes	yes	no
		14	57	male	ALS	yes	yes	no
		15	70	male	ALS	yes	yes	no
		16	74	female	ALS	yes	yes	no
		17	68	male	ALS	yes	yes	no
		18	71	female	ALS	yes	yes	no
