# Supplementary material for: Reconditioning the Neurogenic Niche of Adult Non-human Primates by Antisense Oligonucleotide-Mediated Attenuation of TGFβ Signaling
Source: Neurotherapeutics. 2021 Apr 15;18(3):1963–79. doi: 10.1007/s13311-021-01045-2 (PMC8609055; doi:10.1007/s13311-021-01045-2)

A)

Cellline: ReNcell CX

WB Loading Pattern

Lane	M	1	2	3	4	5	6	7
Sample	Marker	Untreated control	TGFβ 50ng/mL 8d	TGFβ 50ng/mL 12d	Scrambled 10μM	NVP-13 10μM	Scrambled 10μM + TGFβ 50ng/mL	NVP-13 10μM + TGFβ 50ng/mL

B)

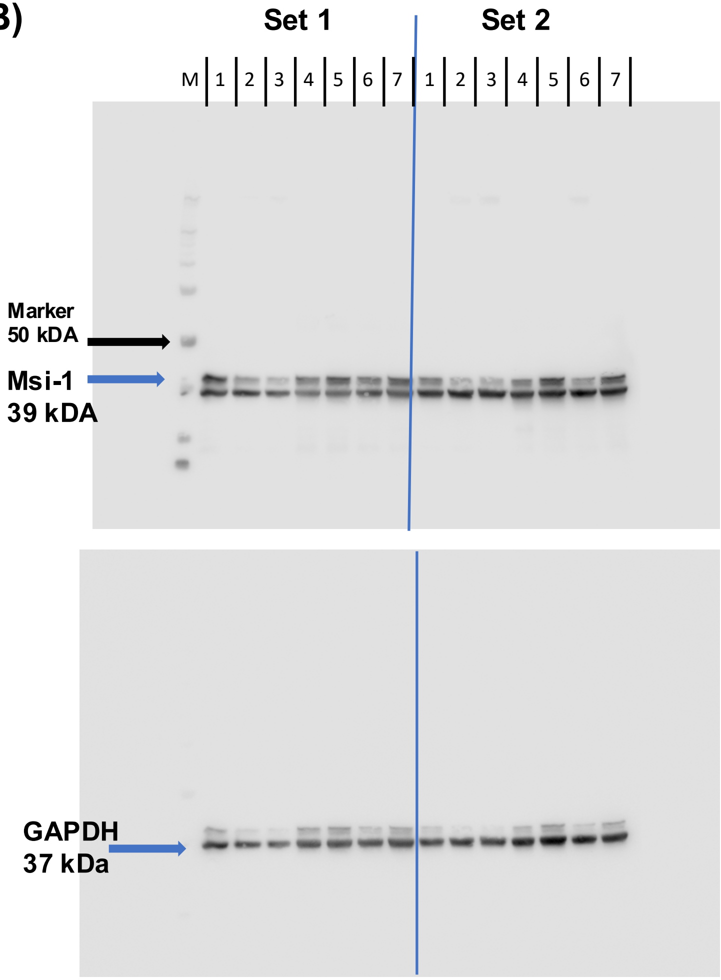

C)

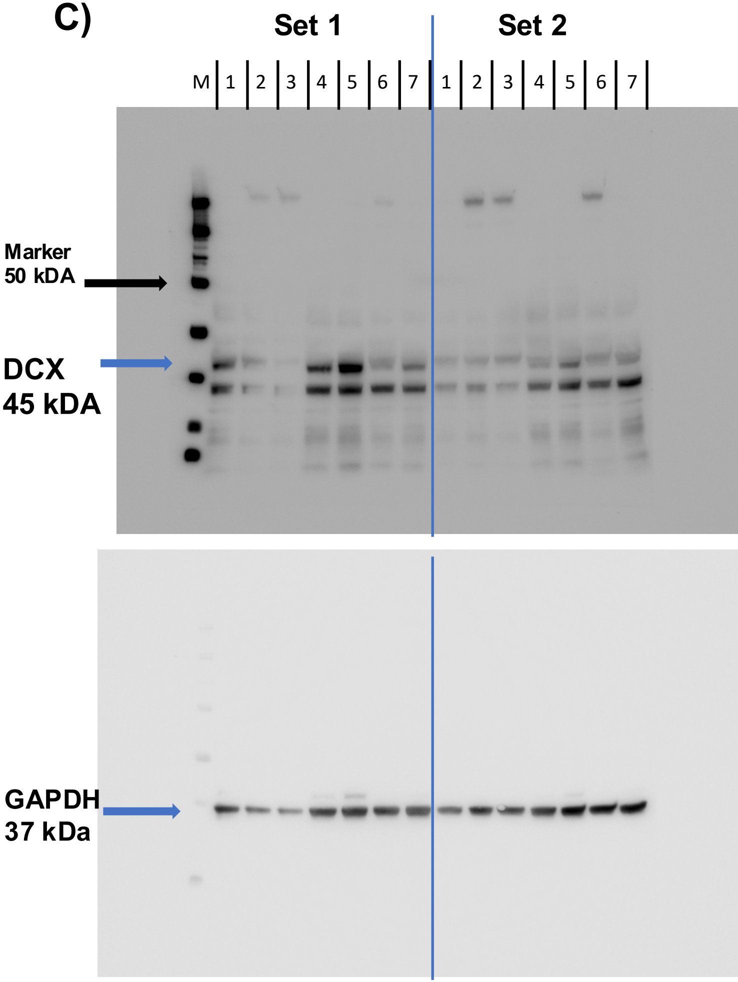

Supplement: Supplementary file 28 — Supplementary file28 (PDF 3419 KB) [file 13311_2021_1045_MOESM28_ESM.pdf]
