# Supplementary material for: Reconditioning the Neurogenic Niche of Adult Non-human Primates by Antisense Oligonucleotide-Mediated Attenuation of TGFβ Signaling
Source: Neurotherapeutics. 2021 Apr 15;18(3):1963–79. doi: 10.1007/s13311-021-01045-2 (PMC8609055; doi:10.1007/s13311-021-01045-2)
Supplement: Supplementary file 29 — Supplementary file29 (PDF 529 KB) [file 13311_2021_1045_MOESM29_ESM.pdf]

	Western Blot			
	**Primary Antibody**	**Dilution**	**Company**	**Order Number**
In vitro analysis/human post-mortem tissue	BMP4	1:500	Genetex	100874
	CTGF	1:2000	Abcam	ab6692
	ColIV	1:2000	Abcam	ab6586
	DCX	1:1000	Cell Signaling	4604
	FN	1:1000	Abcam	ab23750
	FoxO4	1:1000	Abcam	ab128908
	pFoxO4	1:500	Abcam	ab79188
	GAPDH	1:2000	Cell Signaling	CS8884s
	MEF2A-C	1:1000	Abcam	ab64644
	pMEF2AS408	1:2000	Cusabio	CSB-PA000728
	Msi-1	1:2000	Abcam	ab52865
	Pai-1	1:2000	Cell Signaling	11907
	pSmad2	1:2000	Cell Signaling	Cs3104s
	pSmad3	1:1000	Cell Signaling	Cs5920
	pSmad1/5/9	1:1000	Cell Signaling	Cs9511
	TGFβ-RII	1:400	Aviva	ARP447743 T100
	**Immunocytochemistry**			
	**Primary Antibody**	**Dilution**	**Company**	**Order Number**
	TGFβ-RII	1:50	Millipore	06-227
	**Secondary Antibody**	**Dilution**	**Company**	**Order Number**
	Cy3 goat-anti-rabbit	1:1000	Life Technologies	A10520
In vivo analysis monkey tissue	**Western Blot**			
	**Primary Antibody**	**Dilution**	**Company**	**Order Number**
	ColIV	1:2000	Abcam	ab6586
	CTGF	1:1000	Abbiotec	251261
	DCX	1:1000	Cell Signaling	4606S
	FN	1:500	Abcam	ab6328
	FoxO3	1:500	Novus	ABIN4312379
	pFoxO3	1:500	Antikörper-Online	ABIN2269245
	GFAP	1:5000	BioLegend	837204
	Glypican-2	1:1000	Biozol	LS-C473128-100
	GAPDH	1:2000	Cell Signaling	CS8884s
	Iba1	1:500	Abcam	ab5076
	MEF2A-C	1:1000	Abcam	ab64644
	Msi-1	1:750	OriGene	Ta502254
	NCAM	1:1000	Covalab	mab72062
	NeuN	1:2000	Abcam	ab177487
	NG2	1:3000	Merck	AB5320
	Smad2/3	1:1000	Cell Signaling	8828S
	pSmad2/3	1:1000	Cell Signaling	8685S
	Sox-2	1:500	Covalab	Pab75795
	TGFβ-RII	1:500	Biorbyt	Orb214665
	**Immunofluorescence**			
	**Primary Antibody**	**Dilution**	**Company**	**Order Number**
	DCX	1:400	Cell Signaling	4604S
	Sox-2	1:500	Abcam	ab97959
	**Secondary Antibody**	**Dilution**	**Company**	**Order Number**
	Cy3 goat-anti-rabbit	1:2000	Life Technologies	A10520
